# Rational Engineering of Patchoulene Synthase from *Pogostemon cablin* for Enhanced Patchoulene Production

**DOI:** 10.3390/ijms262010187

**Published:** 2025-10-20

**Authors:** Wei Ma, Xiukun Wan, Ge Yao, Fuli Wang, Hui Jiang

**Affiliations:** State Key Laboratory of NBC Protection for Civilian, Beijing 102205, China; weima0224@163.com (W.M.);

**Keywords:** patchoulene, patchoulene synthase, enzyme engineering, semi-rational design, terpenoid production, microbial factory, *Pogostemon cablin*

## Abstract

Patchoulene, the characteristic sesquiterpene of patchouli essential oil, is highly valued in the perfume industry for its distinctive woody note and fixative properties. Beyond its olfactory applications, patchoulene has demonstrated promising biological activities, including anti-inflammatory, antimicrobial, and neuroprotective effects. Current production relies mainly on extraction from *Pogostemon cablin* plants, which requires long growth cycles (≥8 months), exhibits low yields, and imposes significant environmental constraints. To overcome these limitations, this study aimed to enhance the Whole-cell yield of patchoulene synthase (PcPTS) through structure-informed protein engineering. A semi-rational design approach was employed, combining homology modeling, molecular docking, evolutionary analysis, and molecular dynamics simulations to identify functional residues within the enzyme active site. Ala-scanning mutagenesis highlighted Thr532 as essential for catalytic activity, and coevolutionary analysis indicated synergistic effects between Phe456 and Thr532. Site-directed mutagenesis was conducted to generate single (F456M, T532Y) and double (F456M/T532Y, designated M2) mutants. The double mutant M2 showed a 3.62-fold increase in patchoulene production compared to the wild-type enzyme. In silico analyses suggested that the enhanced performance of M2 originates from improved substrate positioning, reduced structural flexibility, and strengthened molecular interactions, collectively contributing to a lower energy barrier for catalysis. This study provides an effective strategy for the rapid optimization of terpenoid synthases and facilitates the development of microbial cell factories for sustainable and high-yield production of plant-derived terpenoids.

## 1. Introduction

Patchoulene, the principal bioactive constituent of patchouli oil [1,2], Recent studies have further revealed its promising pharmacological potential in anti-inflammatory, antimicrobial, and neuroprotective applications [3]. However, conventional plant extraction is constrained by the lengthy cultivation cycle of *Pogostemon cablin* (≥8 months), low in planta patchoulene content, and high environmental resource consumption, all of which hinder its ability to meet market demand. In comparison, Saccharomyces cerevisiae is a well-established microbial chassis featuring a well-defined mevalonate pathway and a mature genetic toolkit, providing a scalable platform for patchoulol biosynthesis. Nonetheless, the intrinsic limitations of natural sesquiterpene synthases—such as low Whole-cell yield and product promiscuity—have long restricted heterologous titers to the milligram level, representing a major bottleneck for industrial production.

This study focuses on patchoulene synthase, the principal enzyme catalyzing patchoulene biosynthesis, whose low expression level and insufficient catalytic activity in Saccharomyces cerevisiae significantly limit fermentation efficiency. To address these challenges, we developed an integrated strategy combining semi-rational design with molecular dynamics (MD) simulations. Specifically, the three-dimensional structure of PcTPS predicted by AlphaFold2 was utilized to precisely define the farnesyl diphosphate (FPP) binding pocket and catalytic active site. Key residues involved in substrate binding and carbocation rearrangement were systematically investigated to construct a virtual saturation mutagenesis library. Subsequent microsecond-scale MD simulations enabled quantitative evaluation of enzyme–substrate complex conformational stability, binding free energy (Δ*G*_bind_) and intermediate transition pathways, thereby synergistically enhancing substrate affinity and catalytic specificity. This approach successfully generated an optimized PcTPS variant and established a rational engineering paradigm—“phylogenetic mining–computational design–dynamic mechanism validation”. Our work provides a methodological framework for efficient patchoulene biosynthesis and for designing terpene synthases with tailored substrate channels.

## 2. Results and Discussion

### 2.1. Structural Basis and Functional Role of THR532 in PcTPS Catalysis

**Figure 1:**
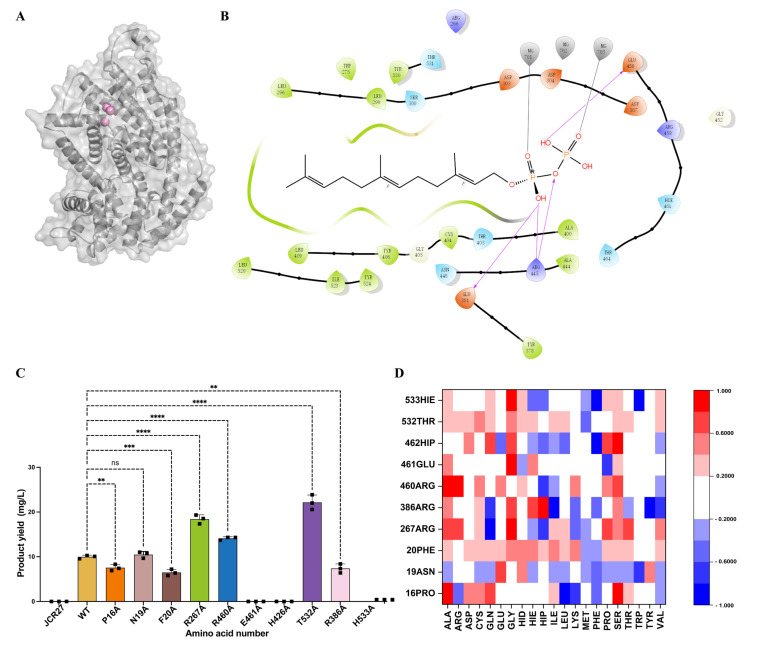
Structural and functional characterization of PcPTS. (**A**) Wild-type PcPTS structure with metal ion coordination; (**B**) Alanine scanning of active pocket residues in patchoulene synthase and the resulting patchoulene yield plot; (**C**) Patchoulene production in alanine-scanning mutants of active-site residues in Patchoulene synthase; **: Significant difference (e.g., *p* < 0.01); ***: Etremely significant difference (e.g., *p* < 0.001); ****: Highly significant difference (e.g., *p* < 0.0001); ns: Not significant; (**D**) Residue Mutation Heatmap of Patchoulene Synthase.

The three-dimensional structure of wild-type (WT) PcTPS was constructed using AlphaFold2, with three Mg^2+^ ions explicitly incorporated at the catalytic center [4]. These metal cofactors play a critical role in stabilizing the enzyme–substrate complex and ensuring correct substrate orientation and polarization within the active site. As shown in Figure 1A, the Mg^2+^ ions form a highly coordinated cluster that not only maintains the overall structural integrity of the enzyme but also provides the electrostatic microenvironment necessary for catalysis. This arrangement is consistent with the catalytic mechanisms of class I terpene synthases, which typically rely on divalent metal ions to activate substrates and stabilize transition states.

To investigate substrate recognition, molecular docking simulations of farnesyl diphosphate (FPP) were performed. Docking results revealed a dense network of interactions surrounding the substrate, including hydrophobic contacts, electrostatic stabilization from positively charged residues, and coordination with Mg^2+^ ions, collectively ensuring precise FPP positioning during catalysis. This structural organization highlights PcTPS’s dual dependence on side-chain chemical complementarity and metal ion synergy for effective substrate recognition [5].

Further analysis of residues within a 5 Å radius of FPP allowed mapping of the local microenvironment of the active site (Figure 1B). A cluster of residues potentially critical for substrate binding and catalysis was identified. To quantitatively assess their functional contributions, alanine-scanning mutagenesis was conducted, systematically evaluating impacts on substrate affinity and product formation. This approach provides direct insight into the importance of catalytic residues and structural redundancy within the active site [6].

Mutagenesis data (Figure 1C) indicated that most alanine substitutions had minor to moderate effects on Patchoulene production, reflecting the structural robustness of PcTPS. In contrast, mutation of THR532 resulted in a significant increase in product yield, underscoring its pivotal catalytic role. This gain-of-function effect suggests that THR532 may modulate substrate orientation or stabilize the transition state. Mutations of other residues, such as ASN219 and HIS533, did not significantly affect activity, implying a primarily structural support role or non-essential participation in the catalytic cycle.

The functional importance of THR532 is further emphasized by its spatial proximity to the catalytic metal cluster and substrate. It likely forms hydrogen bonds or dipole interactions with the FPP pyrophosphate group to optimize substrate geometry, and may also induce local conformational adjustments to facilitate catalytic residue positioning or charge distribution. Therefore, the observed activity enhancement in THR532 mutants may reflect improved catalytic geometry or a reduced reaction energy barrier [7]. Mg^2+^ ions polarize the substrate and stabilize negatively charged intermediates during phosphoryl transfer or isoprenyl chain elongation; the enhanced activity in the THR532 mutant likely arises from cooperative interactions between this residue and the metal cluster, promoting efficient catalysis. In contrast, ASN219 and HIS533 appear to play passive structural roles, maintaining protein stability without directly participating in chemistry. These findings underscore the robustness of PcTPS catalysis, where certain active site residues serve primarily structural functions rather than mediating chemical transformation [8].

To evaluate the engineering potential of THR532, virtual saturation mutagenesis was performed. Computational predictions suggested that specific substitutions at this position could further optimize substrate binding and catalytic turnover (Figure 1D).

### 2.2. Evolutionary Coupling and Mutagenesis Reveal Key Residue Pairs for Catalytic Enhancement in PcTPS

**Figure 2:**
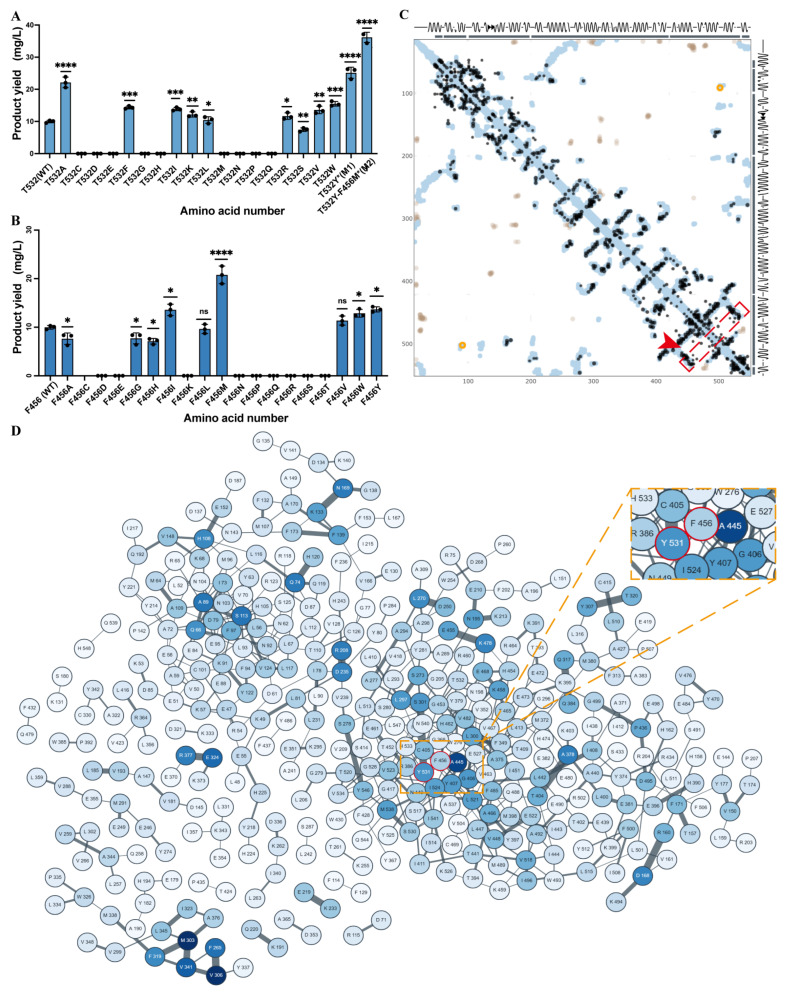
Functional role of key PcPTS residues. (**A**) Patchoulene production in wild-type (WT), *: Significant difference (e.g., *p* < 0.05); **: Significant difference (e.g., *p* < 0.01); ***: Etremely significant difference (e.g., *p* < 0.001); ****: Highly significant difference (e.g., *p* < 0.0001); T532 saturation mutagenesis, and mutant strains M1 (T532Y) and M2 (F456M/T532Y); (**B**) Patchoulene production in wild-type (WT) and F456 saturation mutagenesiss; *: Significant difference (e.g., *p* < 0.05); ****: Highly significant difference (e.g., *p* < 0.0001); ns: Not significant; (**C**) spatial contact map of the PHE456-THR532 residue pair; Red box: Delineates the region containing mutation sites; Red arrow: Directs attention to critical target regions requiring focused attention; (**D**) cooperative interaction network analysis of the PHE456-THR532 residue pair.

Experimental validation confirmed that THR532 consistently yielded higher activity than the WT, highlighting its importance as a rational design target (Figure 2A). In summary, integrating structural modeling, molecular docking, and mutational analysis elucidated the catalytic mechanism of PcTPS and established THR532 as a key residue for functional enhancement, likely by stabilizing the enzyme–substrate complex and facilitating efficient chemical rearrangements. This insight provides a solid theoretical foundation for engineering PcTPS variants with improved Whole-cell yield and broader biotechnological applications.

Evolutionary coupling analysis of PcTPS using the EvCouplings platform revealed a strong co-evolutionary relationship between PHE456 and THR532. A prominent high-signal region between these residues indicates high evolutionary correlation (Figure 2C), reflecting selective pressure to maintain critical interactions essential for enzyme stability and function, thereby highlighting their potential functional interdependence. Structural mapping (Figure 2D) further supported this finding, demonstrating spatial proximity between PHE456 and THR532 that suggests formation of a cooperative interaction network crucial for both catalytic activity and structural integrity.

Structural mapping (Figure 2B) further corroborated this finding: PHE456 and THR532 are spatially proximal, suggesting they may form a cooperative interaction network essential for catalytic activity and structural integrity. Saturation mutagenesis experiments confirmed the functional significance of this residue pair, as individual substitutions markedly enhanced catalytic yield. Specifically, the F456M mutation increased product yield by 1.87-fold (Figure 2B), while the T532Y variant showed a 1.83-fold improvement (Figure 2A). These results indicate direct involvement of both residues in catalysis, potentially through optimization of substrate binding, modulation of active site flexibility, or enhancement of local hydrophobic/electrostatic interactions [5]. The F456M mutation introduces a more flexible methionine side chain while retaining hydrophobicity, likely improving spatial accommodation of the substrate. The T532Y mutation introduces a bulkier aromatic side chain, which may enhance the hydrophobic environment or regulate conformational dynamics to favor catalysis. Notably, the double mutant M2 (F456M/T532Y) exhibited a synergistic effect, achieving a product yield of 36.12 mg/L—3.62-fold higher than WT—far exceeding the individual gains of single mutants (Figure 2A). This result indicates functional synergy between PHE456 and THR532.

Mechanistically, PHE456 likely resides within a hydrophobic pocket, directly interacting with the isoprenoid substrate to stabilize orientation or shield reactive intermediates. THR532, closer to the catalytic metal ions and pyrophosphate binding region, may assist in substrate coordination or mediate local conformational changes. Introduction of methionine and tyrosine alters the spatial and electronic environment of the active site, optimizing substrate affinity and catalytic turnover. The observed synergy in the M2 mutant suggests that simultaneous modification of spatially adjacent residues can induce favorable active site rearrangements, enhancing Whole-cell yield. Integration of evolutionary coupling analysis with experimental validation highlights the value of targeting co-evolved residues in enzyme engineering [9]. This study demonstrates that computationally identified co-evolved residue pairs, such as PHE456–THR532, serve as effective hotspots for functional enhancement. The combination of evolutionary information and mutational screening not only confirms functional connectivity but also provides a rational strategy for enzyme design, with broad applicability in industrial biotechnology to improve Whole-cell yield and product yield.

### 2.3. Altered Binding Mode and Substrate Stabilization in the M2 Mutant

Molecular docking is a widely used computational method to predict ligand–protein interaction patterns at atomic resolution, capturing binding geometries and energetic preferences [10,11,12]. By systematically generating ligand conformations and ranking them based on binding free energy, docking identifies thermodynamically optimal binding modes [13], offering critical insights into molecular recognition mechanisms. This approach is particularly valuable in enzyme engineering and drug design [14,15]. In this study, molecular docking was used to compare FPP binding in WT and M2 PcTPS complexes.

**Figure 3:**
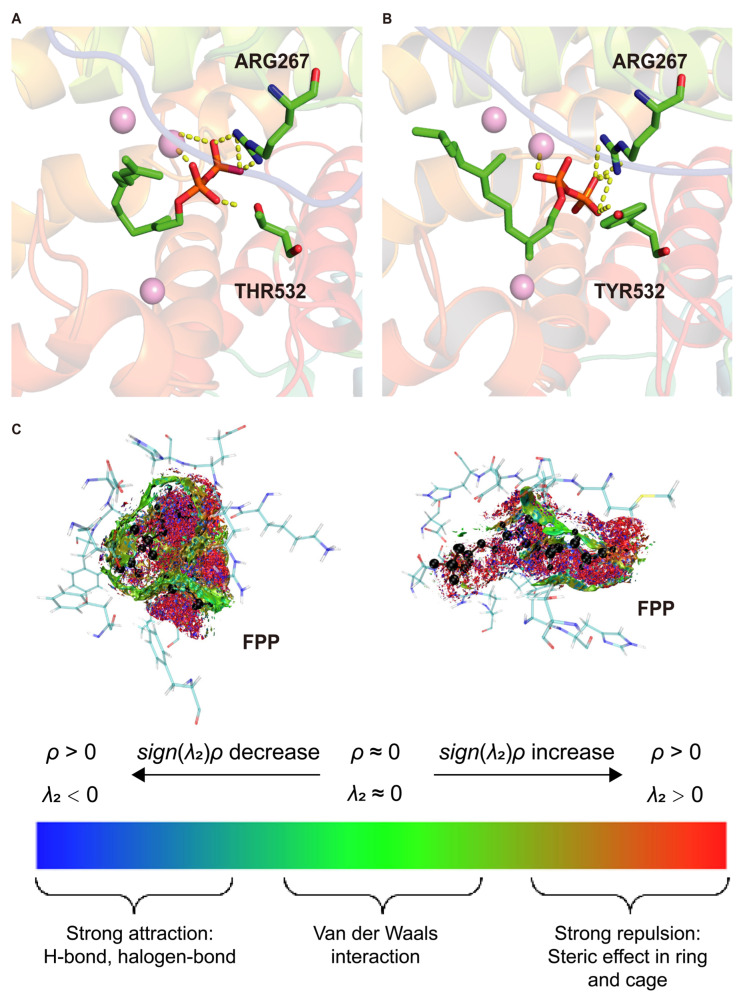
Docking complexes and interaction analysis of wild-type PcPTS and the M2 (F456M/T532Y) mutant. (**A**) Docking complex of the wild type (WT); (**B**) docking complex of the M2 (F456M/T532Y) mutant; (**C**) analysis of noncovalent interactions (aNCI) in the complexes of the WT and M2 mutant.

Docking results revealed significant differences in FPP binding conformations between WT and M2 complexes. In the WT (Figure 3A), residues such as ARG267 and THR532 formed a stable interaction network with FPP and Mg^2+^ ions, with salt bridges between Mg^2+^ and the pyrophosphate group playing a critical anchoring role. In the M2 complex (Figure 3B), the THR532→TYR mutation likely alters steric and hydrogen-bonding interactions, weakening the original salt-bridge network. This reduction in electrostatic constraints may allow FPP to adopt a more rigid and uniform orientation within the mutated active site, promoting preorganization favorable for catalysis.

To further investigate the underlying noncovalent interaction mechanisms, average noncovalent interaction (aNCI) analysis was performed on MD trajectories (Figure 3C). This method visualizes weak interactions such as van der Waals forces, hydrogen bonds, and steric repulsion based on electron density (ρ) and the second eigenvalue (λ_2_) of the Hessian matrix: λ_2_ < 0 and ρ > 0 indicate attractive interactions, whereas λ_2_ > 0 and ρ > 0 indicate repulsion. In the WT–FPP complex, extensive red regions around the pyrophosphate and isoprenoid chain indicate strong steric repulsion and suboptimal substrate accommodation. Sparse and dispersed green van der Waals clouds suggest weak and poorly organized stabilizing interactions, consistent with elevated RMSD values, indicating that the WT active site cannot maintain a catalytically favorable binding conformation.

In contrast, the M2–FPP complex exhibited dense green and blue interaction clouds surrounding the FPP backbone and pyrophosphate tail, reflecting strong van der Waals and polar attractions (including hydrogen bonds). Red repulsive regions were reduced and primarily located at the periphery, indicating that the M2 mutations alleviated steric clashes and reshaped the binding pocket to better accommodate the substrate [16]. This optimized interaction pattern enhances spatial and electrostatic complementarity, resulting in a more energetically favorable and geometrically stable substrate-binding conformation. Such stabilization likely reduces conformational entropy loss upon binding and facilitates transition state stabilization, both critical for efficient catalysis.

Collectively, molecular docking and aNCI analyses indicate that the M2 mutations reconstruct the active site, minimize steric hindrance, and strengthen noncovalent interactions, thereby significantly stabilizing substrate binding. These structural and energetic improvements promote enzyme–substrate complexes toward a transition-state geometry, enhancing overall Whole-cell yield.

### 2.4. Global Conformational Changes and Dynamic Properties

**Figure 4:**
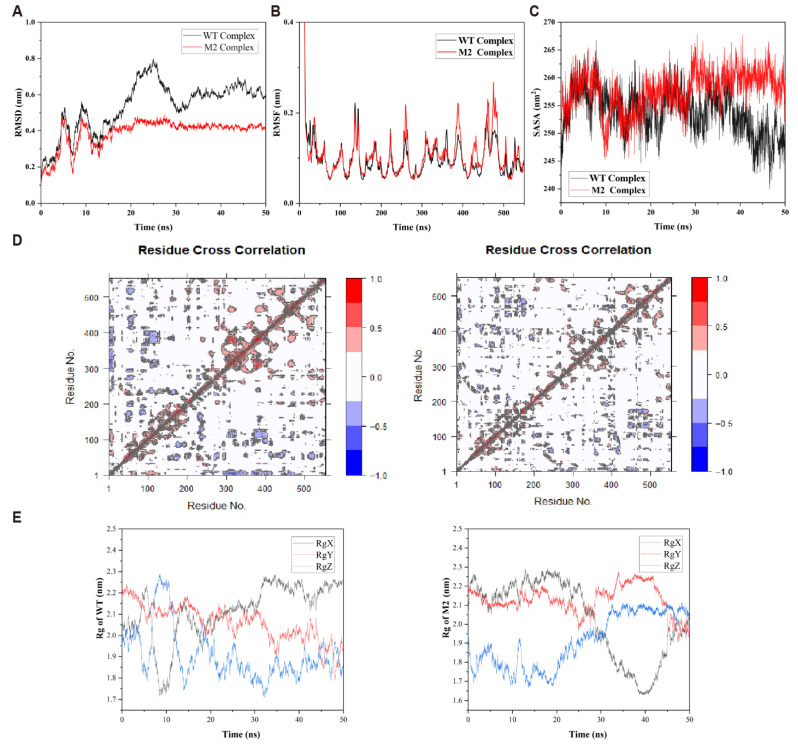
Molecular dynamics trajectory analyses. (**A**) RMSD analysis; (**B**) RMSF analysis; (**C**) SASA analysis; (**D**) differential contact correlation matrix (DCCM) of the wild type (WT) and the M2 (F456M/T532Y) mutant; (**E**) radius of gyration (Rg) analysis of the wild type (WT) and the M2 (F456M/T532Y) mutant.

To investigate the dynamic consequences of the altered binding modes, 50 ns all-atom molecular dynamics (MD) simulations were performed, and the root-mean-square deviation (RMSD) of FPP relative to the protein backbone was analyzed (Figure 4A). RMSD provides a measure of substrate-binding conformational stability during simulation and is an important indicator of binding robustness [17]. In the M2–FPP complex, the substrate exhibited high spatial stability, with RMSD values consistently near 0.2 nm and minimal fluctuations, indicating tight confinement of FPP within the optimized binding pocket. In contrast, the WT–FPP complex showed larger RMSD fluctuations (0.25–1.0 nm), suggesting a dynamic and unstable binding mode with frequent deviations from the initial orientation. Trajectory analysis revealed repeated reorientation of the FPP pyrophosphate group in WT, disrupting critical electrostatic and hydrogen-bond interactions with surrounding residues and Mg^2+^ ions. This conformational instability likely hinders formation of catalytically competent states and increases entropic costs during transition-state formation, reducing Whole-cell yield [18].

The enhanced substrate stability in M2 likely arises from several factors: first, active site mutations improve shape complementarity between the binding pocket and FPP, strengthening van der Waals interactions and spatial matching; second, mutations may remodel the hydrogen-bond network around the pyrophosphate tail, enhancing electrostatic anchoring; additionally, the reduced conformational freedom of the polarized substrate indicates a more rigid electrostatic environment, minimizing entropic penalties during transition-state formation. This increased conformational stability not only lowers chemical barriers but also ensures precise alignment of reactive groups, facilitating efficient bond rearrangement. Thus, the lower RMSD in M2 reflects functional optimization of the enzyme–substrate complex and correlates with higher Whole-cell yield.

To further assess the impact of mutations on protein conformational stability and dynamics at the residue level, root-mean-square fluctuation (RMSF) analysis of the backbone residues was performed for WT and M2 (Figure 4B). RMSF quantifies the deviation of each residue from its average position during MD simulation [9], providing a direct measure of local flexibility and motion trends, and identifying flexible loops, stable cores, and mutation-sensitive regions [10,11,19]. Over 50 ns, the overall RMSF profiles of WT and M2 were broadly similar, indicating comparable global folding and secondary structure dynamics. However, significant local differences were observed, particularly near the mutation sites and adjacent structural elements. In these regions, M2 exhibited markedly lower RMSF peaks, suggesting increased local rigidity. This reduction in flexibility implies the introduction of stabilizing intramolecular interactions, such as hydrogen bonds, hydrophobic packing, or π–π stacking, effectively locking conformations in place. Notably, regions spatially distant from the mutation sites, including portions of the substrate-binding pocket and catalytic center, also showed decreased RMSF in M2, indicating distal structural rearrangements likely propagated from the mutations. Such “remote rigidification” is frequently observed in highly efficient natural enzymes and rationally designed mutants, highlighting non-local responses of protein structure to point mutations. Overall, the smoother RMSF profile in M2, with fewer sharp peaks, suggests more focused vibrational modes, more evenly distributed energy, and occupation of lower-energy conformational states, consistent with RMSD analysis and emphasizing the enhanced rigidity and global stability of the mutant protein.

To further examine the effect of mutations on protein hydrophobicity and surface properties, solvent-accessible surface area (SASA) was calculated from MD trajectories (Figure 4C). SASA represents the total surface area accessible to a solvent probe and correlates with folding compactness, structural stability, and burial of hydrophobic cores [13]. Lower SASA values generally indicate more compact conformations with hydrophobic cores effectively shielded from solvent [14]. As shown in Figure 4C, both WT and M2 rapidly reached equilibrium early in the simulation, maintaining stable SASA values. However, while WT averaged ~120 nm^2^, M2 exhibited a significantly lower average SASA of ~115 nm^2^ with reduced fluctuation, indicating a more compact overall conformation and better shielding of the hydrophobic core. This surface property alteration enhances overall protein stability and may tighten the substrate-binding pocket. These observations are consistent with prior RMSD and RMSF analyses.

To further characterize the effect of the F456M/T532Y double mutation on protein dynamics, dynamic cross-correlation matrices (DCCM) were generated from the 50 ns MD trajectories of WT and M2 (Figure 4D). In WT, extensive positive (red) and negative (blue) correlations were observed, particularly between residues 200–300 and 400–500, suggesting long-range coordinated and anti-correlated motions across structural domains. This pattern indicates a flexible, highly coupled architecture with extensive residue communication. In contrast, M2 displayed markedly attenuated correlation patterns, with weaker correlated blocks, reflecting redistribution of structural flexibility toward local rigidification. Notably, regions surrounding the mutation sites (residues 450–540) exhibited reduced cross-correlation with other domains, supporting the hypothesis that the mutations stabilize the active site environment and reduce unnecessary conformational fluctuations. This local stabilization likely contributes to substrate pre-organization and the observed increase in Whole-cell yield in M2. Together with RMSD and RMSF data, these results indicate that the double mutation improves the balance between rigidity and flexibility, reinforcing key catalytic regions while maintaining global adaptability required for substrate turnover.

To elucidate the impact of the F456M/T532Y double mutation on the global conformational integrity of the enzyme, we performed 50 ns molecular dynamics simulations and analyzed the radius of gyration (Rg) for both the wild-type (WT) and M2 complexes [12]. The Rg, a measure of the mass-weighted root-mean-square distance of atoms from the protein’s center of mass, serves as a metric for quantifying the compactness of the tertiary structure. A lower and more stable Rg trajectory is indicative of a structurally compact, rigid, and thermodynamically stable protein conformation.

Our analysis revealed different dynamic behaviors between the WT and M2 proteins. The WT enzyme exhibited a fluctuating Rg profile, oscillating between 2.2 nm and 2.3 nm, which signifies conformational flexibility. This instability was also observed in the axial components of Rg (RgX, RgY, and RgZ). We identified anti-correlated oscillations between the RgX and RgZ components, suggesting anisotropic motions, such as domain reorientations, that destabilize the protein’s overall shape.

In contrast, the M2 variant displayed a stabilized dynamic signature. Its total Rg trajectory was characterized by a mean value of approximately 2.2 nm and showed minimal deviation over the simulation period. This observation indicates that the double mutation induces a more compact and structurally coherent tertiary fold. Furthermore, an attenuation of fluctuations was observed in the axial components; the RgX, RgY, and RgZ values remained constrained to minor thermal vibrations around their respective means, confirming the suppression of large-scale conformational rearrangements.

Conversely, the M2 mutation enforces a state of structural rigidity, locking the enzyme into a compact and functionally optimized conformation. The stabilization of the axial Rg components indicates a restriction of the protein’s accessible conformational space, suppressing non-productive, large-amplitude motions. In conclusion, the Rg analysis demonstrates that the engineered mutations induce a global structural rigidification, providing a physical basis for the observed enhancement in catalytic activity.

Integrating RMSD, RMSF, SASA, and Rg analyses, M2 demonstrates a more compact and cohesive conformational profile. This increased structural compactness not only stabilizes the protein overall but also optimizes the substrate-binding microenvironment, facilitating more efficient FPP binding and enhanced catalytic performance. These findings provide compelling structural evidence supporting the semi-rational design of enzymes with improved catalytic activity.

### 2.5. Principal Component Analysis (PCA) and Free Energy Landscape (FEL)

**Figure 5:**
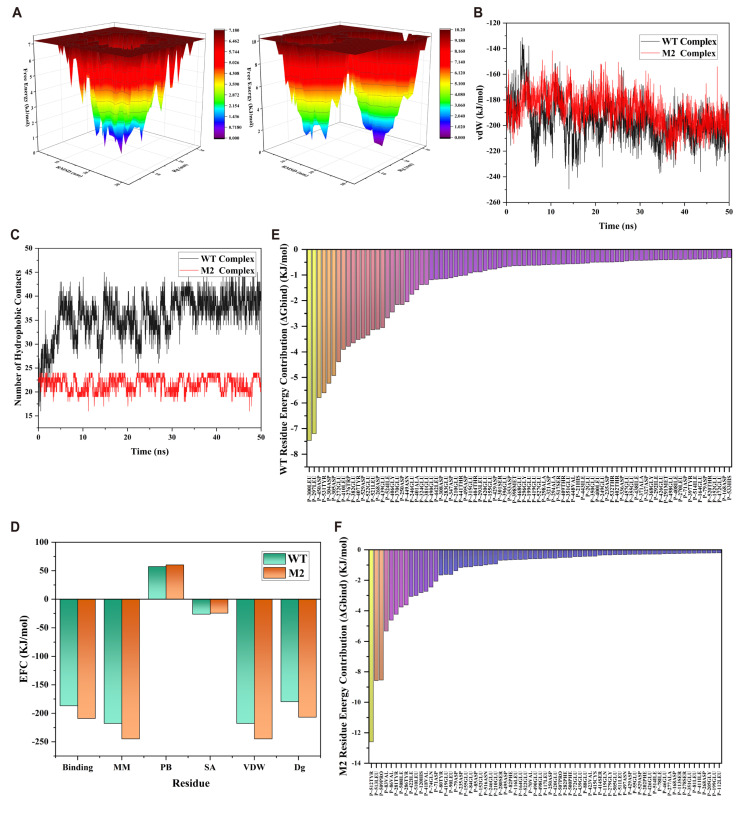
Molecular dynamics binding energy analyses. (**A**) Free energy landscape (FEL) analysis; (**B**) van der Waals interaction energy analysis; (**C**) SASA analysis; (**D**) MM/PBSA analysis; (**E**) per-residue binding free energy contribution in WT; (**F**) per-residue binding free energy contribution in M2.

Principal Component Analysis (PCA) is a statistical approach widely used to reduce the dimensionality of high-dimensional MD trajectories, allowing identification of dominant motions and amplitudes in protein dynamics [17]. At the molecular level, complex conformational changes can be described by a set of orthogonal eigenvectors, or principal modes of motion, with the first two principal components (PC1 and PC2) typically capturing the majority of total variance [18]. These components reveal key factors driving conformational transitions. In this study, 50 ns MD trajectories of WT and M2 protein–FPP complexes were analyzed by constructing a covariance matrix of atomic fluctuations, which was then diagonalized to extract PC1 and PC2. Two-dimensional projections in PC1–PC2 space were generated to visualize the amplitude and distribution of conformational sampling (Figure 5A).

The WT system exhibited a widely dispersed distribution of trajectory points in PC1–PC2 space, forming multiple independent clusters. This indicates frequent conformational rearrangements during the simulation, exploring diverse dynamic modes. Such high-amplitude, non-convergent motion reflects greater structural flexibility and conformational freedom, which may destabilize the catalytic pocket geometry and reduce substrate-binding stability. In contrast, the M2 mutant displayed a highly concentrated distribution of points, forming a compact cluster indicative of “conformational restriction”. This suggests the mutant predominantly adopts a single dominant conformational state over time, with restricted motion and a steeper energetic landscape. These observations are consistent with FEL analysis, which revealed a deep, localized energy minimum in M2, indicating enhanced thermodynamic preference for catalytically favorable conformations. Moreover, PC1 and PC2 account for a higher proportion of total variance in M2, suggesting reduced redundant motion and increased dominance of primary dynamic modes. This compression of conformational space and increased principal component contribution is characteristic of “structural convergence”, commonly observed in highly efficient enzymes, promoting precise substrate recognition and effective catalysis.

Overall, PCA indicates two key dynamic features in M2: (1) decreased overall conformational freedom, and (2) increased stability around the dominant conformational state. This dynamic convergence enhances structural stability and maintains optimal geometry of the active site, supporting efficient and selective substrate catalysis. These results are highly consistent with RMSD, RMSF, Rg, and SASA analyses, highlighting the importance of mutation-induced conformational control in functional enhancement.

The Free Energy Landscape (FEL) provides a powerful visualization of protein motion in conformational space and the distribution of energetic states [16]. FEL analysis enables understanding of conformational diversity, energy barrier heights, and stability of dominant states under quasi-equilibrium conditions [20]. In this study, FELs were reconstructed using PC1 and PC2 as reaction coordinates for WT and M2 protein–FPP complexes to reveal thermodynamic mechanisms underlying mutation-induced conformational changes.

The FEL of WT exhibited a typical multi-valley, multi-path topology, with several energy minima of varying depths connected by relatively low barriers. This indicates that the wild-type protein sampled multiple stable or metastable conformations, showing high conformational diversity and accessibility. While such dispersion allows structural adaptability, it also reflects a relatively flat energy surface with high flexibility, potentially leading to frequent transitions into catalytically suboptimal or inactive conformations, thereby impairing substrate recognition and catalytic specificity.

In contrast, the M2 FEL was dominated by a single, deep, and stable energy minimum, forming a highly localized “converged” free energy configuration. The lowest energy basin was tightly clustered near the energy baseline, with minimal peripheral metastable states. This indicates significant compression of conformational space, with the protein predominantly occupying one well-defined, favorable conformation. Notably, large energy barriers separate the global minimum from other high-energy conformations, implying that transitions away from the dominant state require substantial thermodynamic cost, in contrast to WT. This energy landscape reflects two critical thermodynamic consequences of the M2 mutation: (1) steepened energy surface effectively penalizes non-ideal conformations, “filtering out” unfavorable states; (2) deepened and converged energy minimum stabilizes catalytically favorable conformations. Consequently, the M2 mutant maintains high structural fidelity and preferentially resides in a single active conformation even in dynamic conditions. This localized sampling pattern is commonly observed in both highly efficient natural enzymes and rationally designed mutants, reducing active site drift and enhancing spatial precision of substrate binding and catalysis.

In conclusion, FEL analysis further confirms enhanced structural stability and reduced conformational flexibility in M2, revealing that its conformational sampling is more functionally oriented and thermodynamically focused. These insights complement PCA findings and provide a mechanistic basis for improved Whole-cell yield in the M2 mutant.

### 2.6. Hydrogen Bond and van der Waals (vdW) Interactions

To comprehensively evaluate the effects of mutations on the microscopic interactions at the enzyme–substrate interface, we analyzed two key non-covalent interactions—hydrogen bonds (H-bonds) and van der Waals (vdW) forces—between PcPTS and farnesyl pyrophosphate (FPP) during 50 ns MD simulations [21,22]. While individual non-bonded interactions are relatively weak, their cooperative effect is critical for maintaining binding stability, complex formation efficiency, and transition-state stabilization.

As shown in Figure 5B, the M2 mutant maintains a higher average number of H-bonds with FPP throughout the simulation, with smaller fluctuations and overall stability. The H-bond trajectory exhibits periodic breaking and reformation, indicating a dynamic yet robust H-bond network. This suggests that mutations optimize H-bonding patterns within the active site, potentially by introducing new donor/acceptor residues or inducing local conformational rearrangements of pre-existing residues. Enhanced H-bonding not only improves spatial precision of substrate positioning but also stabilizes reaction intermediates, thereby facilitating efficient catalysis.

Correspondingly, vdW interaction analysis reveals a consistent trend. The average vdW interaction energy between enzyme and FPP in M2 is approximately −200 kJ/mol, significantly stronger than −150 kJ/mol in WT, indicating enhanced attractive forces and tighter hydrophobic contacts. These interactions primarily arise from close packing between nonpolar side chains of the active site and hydrophobic regions of FPP. Structural optimization in M2 likely enhances short-range atomic attraction, further stabilizing the complex and improving spatial complementarity. Stronger vdW interactions correspond to a more compact hydrophobic interface, limiting substrate mobility and reducing conformational drift. This observation aligns with previous SASA and Rg analyses, confirming a tighter binding conformation and reduced solvent exposure in M2.

Notably, hydrogen bonding and vdW forces act synergistically rather than independently. The H-bond network provides directional locking, while hydrophobic packing reinforces spatial fixation. Together, they form a geometrically precise and energetically stable catalytic environment. This “dual optimization mechanism” stabilizes transition states and maintains the substrate in a well-aligned, conformationally faithful orientation—features typical of highly efficient enzymes.

In summary, analysis of H-bonds and vdW interactions demonstrates that M2 mutations optimize the enzyme–substrate interface through enhanced non-covalent interactions. The increase in H-bonds and strengthened vdW forces collectively improve substrate affinity, providing molecular-level evidence for the experimentally observed enhancement in M2 Whole-cell yield.

### 2.7. Hydrophobic Interaction Analysis

Hydrophobic interactions are among the most critical non-covalent forces regulating enzyme–substrate recognition and complex stability, playing a central role in maintaining biomolecular conformation and function [23]. This mechanism is particularly important when substrates contain long nonpolar groups, such as FPP [24]. Hydrophobic networks within the active site directly influence substrate binding conformation, residence time, and energy barrier distribution along the reaction coordinate. Therefore, tracking the dynamic evolution of hydrophobic contacts is essential for understanding how mutations reshape the spatial recognition interface and affect binding behavior and catalysis.

As shown in Figure 5C, WT and M2 proteins display marked differences in hydrophobic interactions with FPP during the 50 ns MD simulations. M2 maintains a higher and more stable number of hydrophobic contacts (average 10–12, minimal fluctuation), suggesting a highly stable and reproducible binding interface. This indicates that the M2 active pocket is better optimized to accommodate FPP in a nonpolar environment, effectively suppressing random thermal motion and substrate misalignment.

In contrast, WT exhibits fewer hydrophobic contacts (average 6–8) with periodic fluctuations, reflecting a looser and less stable hydrophobic interface. Such instability may allow the substrate to sample multiple binding conformations, disrupting catalytic alignment and reducing transition-state formation efficiency.

Mechanistically, the enhanced hydrophobic interactions in M2 likely arise from: (1) introduction of additional hydrophobic residues (e.g., Phe, Leu, Val) that directly engage FPP, and (2) local conformational rearrangements that reposition originally solvent-exposed hydrophobic side chains toward the binding pocket, forming a more compact “hydrophobic core”. This structural remodeling increases rigidity and reduces solvent infiltration, providing a nonpolar microenvironment critical for stabilizing the polar pyrophosphate moiety of FPP.

Thermodynamically, the stable and extensive hydrophobic contacts correlate with vdW energy contributions. In M2, more negative vdW energies align with stronger hydrophobic interactions, highlighting the structural–energetic synergy. Optimization at both geometric and energetic levels indicates that the mutation enhances substrate spatial complementarity and overall binding stability.

### 2.8. MM/PBSA Analysis

Molecular mechanics/Poisson–Boltzmann surface area (MM/PBSA) is a widely used semi-empirical method to estimate protein–ligand binding free energies [25]. By decomposing the total free energy into several energetic components, MM/PBSA provides insights into the thermodynamic nature and mechanistic features of enzyme–substrate interactions. In this study, based on 50 ns MD trajectories, the binding affinities between wild-type (WT) or M2 mutant PcPTS and FPP were evaluated using MM/PBSA (Figure 5D). The analyzed energy terms included total binding energy, molecular mechanics energy (MM), polar solvation energy (PB), nonpolar solvation energy (SA), van der Waals (vdW) interactions, and overall free energy change (Δ*G*).

The binding free energy of M2 was calculated as −209.186 kJ/mol, significantly lower than −186.868 kJ/mol for WT, indicating a more thermodynamically stable interaction. Lower Δ*G* typically correlates with higher binding affinity, suggesting that M2 mutations substantially enhance enzyme–substrate stability. The MM energy for M2 was −244.843 kJ/mol versus −217.855 kJ/mol for WT, reflecting stronger internal interactions, including vdW and electrostatics.

vdW interactions are the main driving force in both systems, with M2 showing ~27 kJ/mol more favorable vdW energy than WT, consistent with enhanced hydrophobic contacts and active site packing observed previously. Polar solvation energy (PB) differed slightly (M2: 60.159 kJ/mol; WT: 57.295 kJ/mol), likely due to local charge redistribution and formation of new H-bonds or conformational rearrangements near polar residues; however, its contribution to overall binding affinity is minor. Nonpolar solvation energy (SA) was slightly less favorable in M2 (−24.503 kJ/mol vs. −26.308 kJ/mol for WT), possibly reflecting minor adjustments in solvent exposure due to local pocket compression, but its impact on total Δ*G* is negligible. The final binding free energy Δ*G* integrates all contributions: −206.948 kJ/mol for M2 versus −179.744 kJ/mol for WT, representing an improvement of ~27 kJ/mol. This indicates that the enhanced binding is primarily driven by molecular mechanics interactions, particularly vdW forces, rather than solvation effects.

Overall, MM/PBSA results confirm that the M2 mutations substantially strengthen enzyme–substrate binding via enhanced vdW interactions, providing thermodynamic support for the experimentally observed catalytic improvement.

### 2.9. Per-Residue Binding Free Energy Decomposition

To elucidate the local energetic basis for enhanced substrate binding in M2, per-residue MM/PBSA decomposition was performed. This analysis quantifies each amino acid’s contribution to the overall binding free energy, identifying residues critical for stabilizing the complex. Figure 5E,F show per-residue contributions for WT and M2. In both systems, multiple residues—including hydrophobic residues (Phe, Leu, Val, Ile) and polar/charged residues (Arg, Glu, Tyr)—contribute favorably to binding, highlighting a cooperative mechanism of hydrophobic packing and H-bonding.

In M2, several key residues exhibit stronger negative contributions compared to WT. Notably, residues adjacent to the mutation sites, which were weak or neutral contributors in WT, now display significantly enhanced binding energies. This indicates that mutation-induced local conformational rearrangements activate residues previously on the periphery, allowing them to participate more effectively in substrate stabilization.

Hydrophobic residues (e.g., Leu, Phe) show markedly stronger contributions in M2, suggesting an optimized hydrophobic pocket that enhances substrate encapsulation and complex stability. Some residues not previously major contributors in WT gain significant energetic roles in M2, including one or two negatively charged residues that form new electrostatic interactions, indicating coordinated reorganization of the binding interface and electrostatic environment. Additionally, residues contributing positively (unfavorably) to binding are fewer and weaker in M2 than in WT, reflecting removal of suboptimal interactions and construction of a more energetically favorable binding surface.

In WT, favorable contributions are broadly distributed, producing a “shallow and wide” binding profile, whereas M2 exhibits a “concentrated and deep” pattern, where a few core residues bear stronger negative binding energies, creating an energy-focused structural basis. Such localization of interaction energy likely stabilizes the catalytic pocket and supports functional integrity.

In summary, per-residue free energy decomposition reveals that M2 mutations enhance binding via local pocket reconstruction, strengthened contributions from key residues, and elimination of interfering interactions. These changes cooperatively improve specificity and stability of the M2–FPP complex, explaining the increased affinity and catalytic potential at a residue-level mechanistic basis.

## 3. Materials and Methods

### 3.1. Wild-Type Receptor and Mutant Generation

The three-dimensional structures of the wild-type (WT) and M2 mutant PcTPS were predicted using AlphaFold2 (London, UK) [26]. For each protein, all five pre-trained model weights were used, and the top-ranked model (ranked_0.pdb) with the highest average predicted Local Distance Difference Test (pLDDT) score (>90) was selected for subsequent analysis. To simulate the native catalytic environment, three Mg^2+^ ions were introduced into the active site to stabilize the enzyme–substrate complex.

To identify critical residues, alanine scanning mutagenesis was first performed. Virtual saturation mutagenesis was then conducted for key sites, and the optimal single-point mutant (M1) was identified based on production yield data. The relationship between the M1 site and other residues was investigated using the EvCouplings (Boston, USA and Munich, Germany) platform and Multiple Sequence Alignment via MAFFT (Osaka University, Japan) to identify co-evolving residues. Saturation mutagenesis of a co-evolving residue revealed a secondary site that synergistically enhanced production, yielding the double mutant M2. Mutant construction was performed experimentally using a site-directed mutagenesis kit (Beyotime-QuickMutation Random Mutagenesis Kit, Shanghai, China).

### 3.2. Computational Tools and Structure Optimization

The computational workflow utilized several specialized software packages. Initial protein structures were predicted using the AlphaFold2 [26] model (monomer_ptm_v2) on the Google Colab platform, and evolutionary coupling analysis was performed using the EvCouplings platform and MAFFT. All structure visualization and in silico mutagenesis were conducted using PyMOL v2.5 (Cambridge, MA, USA) [27]. Prior to docking, the selected AlphaFold2 structures underwent an initial refinement through energy minimization using the GROMACS 2024.3 (Stockholm, Sweden; Uppsala, Sweden; Berlin, Germany; and Boulder, CO, USA) [28] package with the CHARMM36 force field. This optimization process employed the steepest descent algorithm for a maximum of 50,000 steps or until the maximum force on any atom was less than 1000 kJ/mol/nm, a step designed to relax the structure and resolve potential steric clashes. Molecular docking was subsequently performed using AutoDock 4.2 [26] with its accompanying AutoDockTools (ADT), while GROMACS 2024.3 was also used for all molecular dynamics simulations. Final data visualization and figure preparation were carried out using PyMOL2.5.x, Matplotlib3.8.x, and Origin2024.

### 3.3. Molecular Docking

The substrate, farnesyl pyrophosphate (FPP), was constructed as a 2D structure in ChemDraw 22.0 to ensure correct atom connectivity, and subsequently converted to a 3D conformation and energy-minimized.

Following AlphaFold2 prediction [29], PyMOL was used for geometric optimization [28], which included removing redundant chains, crystal waters, and non-relevant ligands. Hydrogen atoms were then automatically added. The structures were further prepared for docking using AutoDockTools, which involved adding polar hydrogens and assigning Gasteiger partial charges [27].

A grid of 60 × 60 × 60 points was set, with a spacing of 0.375 Å between points, which corresponds to an actual size of approximately 22.5 × 22.5 × 22.5 Å, was centered on the putative active site of each protein. The Lamarckian Genetic Algorithm (LGA) was employed for the conformational search over 100 independent docking runs. Key LGA parameters included a population size of 150 and a maximum of 2,500,000 energy evaluations.

The resulting poses were clustered based on a root-mean-square deviation (RMSD) tolerance of 2.0 Å. The lowest-energy conformation from the most populated cluster was visually inspected using PyMOL and selected as the most energetically favorable and biologically relevant complex for subsequent simulations.

### 3.4. Molecular Dynamics (MD) Simulation

MD simulations provide a computational approach to study atomic motion by solving Newton’s equations of motion. To assess the stability and dynamics of the selected protein–ligand complexes, 50 ns MD simulations were performed for each system using GROMACS (Stockholm, Sweden; Uppsala, Sweden; Berlin, Germany; Boulder, USA), a high-performance engine for biomolecular simulations [25]. All simulations were repeated independently three times to ensure reproducibility.

### 3.5. Post-MD Analysis

Non-covalent Interaction Analysis

Hydrogen bonds were counted using gmx hbond, and van der Waals interaction energies were obtained from the “LJ-SR: Protein-FPP” term in gmx energy. Hydrophobic contacts were analyzed with custom Python (https://www.python.org/) scripts by identifying nonpolar atom pairs between the protein and FPP.

Trajectory Analysis

The effects of mutations on global protein motions were assessed by generating dynamic cross-correlation matrices (DCCMs). Protein stability was evaluated by calculating the RMSD and RMSF of the backbone and individual residues, the radius of gyration (Rg), and the solvent-accessible surface area (SASA). Principal component analysis (PCA) was performed to extract major motions (PC1 and PC2), and free energy landscapes (FELs) were reconstructed from PCA projections to identify stable states.

Binding Free Energy Calculations

Binding free energies were estimated using the g_mmpbsa tool on 50 ns trajectories, sampling every 100 frames. The total binding free energy (Δ*G*_binding_) was decomposed into molecular mechanics energy (MM), polar solvation (PB), nonpolar solvation (SA), and van der Waals (vdW) contributions.

### 3.6. Construction of Recombinant Plasmids for Wild-Type and Mutant Yeast Expression

Plasmids encoding WT/mutant genes were constructed by Gibson assembly [30] (pYH328 and inserts, 50 °C/30 min), transformed into *E. coli* DH5α with ampicillin selection (100 μg/mL) and sequencing validation; recombinant 2.2 kb fragments (50 bp homology arms, HiFi PCR) were integrated into *S. cerevisiae* JCR27 via LiAc/SS-DNA transformation [31]. Engineered strains were fermented in YPD (1% glucose/galactose, 30 °C, 220 rpm, 24 h) followed by 10% n-decane addition (48 h), with products quantified by GC-MS. Optimization studies in S. cerevisiae JCR27 evaluated pH (4.0–6.0), inoculum (1–5% *v*/*v*), and duration (24–72 h) effects on OD600 and patchoulene production under these conditions.

## 4. Conclusions

In this study, we integrated computational biology with experimental validation to systematically analyze and rationally engineer Patchoulene synthase (PcPTS), achieving a substantial enhancement in both Whole-cell yield and product yield. These efforts lay a crucial foundation for the industrial-scale biosynthesis of Patchoulene as a high-energy liquid biofuel. Low Patchoulene yield is primarily constrained by two factors: insufficient intrinsic catalytic activity of the enzyme and the formation of complex product profiles with numerous by-products, which diminish the yield of the target product. To address the former, we implemented a systematic semi-rational design strategy and obtained significant improvements.

First, through AlphaFold 2 structural modeling and molecular docking analyses, we characterized the catalytic center of PcPTS, highlighting the critical role of three magnesium ions in stabilizing the substrate conformation and facilitating catalysis. Subsequent alanine scanning identified THR532 as a key residue influencing catalytic performance; its mutation markedly increased Patchoulene production, underscoring the necessity of precise interventions at critical active-site residues. Evolutionary coupling analysis further revealed a strong co-evolutionary relationship between PHE456 and THR532. Experimental results showed that single-point mutations F456M and T532Y increased Patchoulene yield by approximately 1.87- and 1.83-fold, respectively, whereas the double mutant M2 (F456M/T532Y) exhibited a pronounced synergistic effect, achieving a 3.62-fold increase relative to the wild type. These findings demonstrate that rational design guided by structural and evolutionary information can effectively overcome the limitations of natural enzyme Whole-cell yield, representing a powerful strategy for the leapwise enhancement of enzyme performance.

Molecular dynamics simulations, free energy calculations, and non-covalent interaction analyses revealed the mechanistic basis for the improved performance of the M2 mutant at the atomic level. The mutations induced structural rearrangements in the active site, enhanced substrate binding stability, optimized the coordination distance between the substrate and catalytic magnesium ions, and lowered reaction energy barriers, thereby favoring transition-state conformations and significantly improving Whole-cell yield.

However, achieving efficient biosynthesis of Patchoulene requires more than just enhanced enzyme activity; issues such as limited catalytic selectivity and by-product formation must also be addressed. Accordingly, this study provides a clear direction for further exploration: detailed investigations into the enzyme’s catalytic mechanism using quantum chemical calculations and reaction pathway analyses are needed to elucidate the formation routes of Patchoulene and other by-products and to identify key regulatory residues. Such insights will enable the precise modulation of the product profile, minimizing by-product formation and further improving Patchoulene yield in a targeted and efficient manner, thereby establishing a solid foundation for industrial application.

In summary, this study not only achieved a highly efficient engineering of PcPTS, substantially increasing terpenoid production, but also established an integrated rational design pipeline combining structural biology, evolutionary analysis, and computational modeling. This strategy can significantly shorten enzyme engineering cycles, improve design success rates, and provide a robust theoretical and technical basis for developing efficient and stable Patchoulene-producing microbial cell factories, thereby accelerating the industrial implementation of Patchoulene as a high-performance biofuel.

## Data Availability

The data from this study are available for sharing. Researchers with a legitimate need may contact the corresponding author to obtain the data.

## References

[1] Chen L., Xie L., Wang L., Zhan X., Zhuo Z., Jiang S., Miao L., Zhang X., Zheng W., Liu T.-M. (2025). Patchoulene epoxide mitigates colitis and hepatic damage induced by dextran sulfate sodium by regulating the colonic microbiota and purine metabolism. Front. Immunol..

[2] Liang Y., Wang F., Song Y., Tang C., Wu R., Feng Q., Han M., Li Y., Chen W., Zhang J. (2024). LC-MS based metabonomics study on protective mechanism of ESWW in cerebral ischemia via CYTC/Apaf-1/NDRG4 pathway. Phytomedicine.

[3] Xu N., Luo H., Li M., Wu J., Wu X., Chen L., Gan Y., Guan F., Li M., Su Z. (2021). β-patchoulene improves lipid metabolism to alleviate non-alcoholic fatty liver disease via activating AMPK signaling pathway. Biomed. Pharmacother..

[4] Hu Z., Liu Y., Huang Y., Yu P. (2025). Advances in the Directed Evolution of Computer-aided Enzymes. Curr. Top. Med. Chem..

[5] Rajakumara E., Abhishek S., Nitin K., Saniya D., Bajaj P., Schwaneberg U., Davari M.D. (2022). Structure and cooperativity in substrate–enzyme interactions: Perspectives on enzyme engineering and inhibitor design. ACS Chem. Biol..

[6] Osuna S. (2021). The challenge of predicting distal active site mutations in computational enzyme design. Wiley Interdiscip. Rev. Comput. Mol. Sci..

[7] Kunka A., Marques S.M., Havlasek M., Vasina M., Velatova N., Cengelova L., Kovar D., Damborsky J., Marek M., Bednar D. (2023). Advancing enzyme’s stability and catalytic efficiency through synergy of force-field calculations, evolutionary analysis, and machine learning. ACS Catal..

[8] Leveson-Gower R.B., Mayer C., Roelfes G. (2019). The importance of catalytic promiscuity for enzyme design and evolution. Nat. Rev. Chem..

[9] Kim D., Noh M.H., Park M., Kim I., Ahn H., Ye D.-y., Jung G.Y., Kim S. (2022). Enzyme activity engineering based on sequence co-evolution analysis. Metab. Eng..

[10] Pujadas G., Vaque M., Ardevol A., Blade C., Salvadó M.-J., Blay M., Fernandez-Larrea J., Arola L. (2008). Protein-ligand docking: A review of recent advances and future perspectives. Curr. Pharm. Anal..

[11] Sahu M.K., Nayak A.K., Hailemeskel B., Eyupoglu O.E. (2024). Exploring recent updates on molecular docking: Types, method, application, limitation & future prospects. Int. J. Pharm. Res. Allied Sci..

[12] Rehman A.U., Khurshid B., Ali Y., Rasheed S., Wadood A., Ng H.-L., Chen H.-F., Wei Z., Luo R., Zhang J. (2023). Computational approaches for the design of modulators targeting protein-protein interactions. Expert Opin. Drug Discov..

[13] Murcko M.A. (1995). Computational methods to predict binding free energy in ligand-receptor complexes. J. Med. Chem..

[14] Honarparvar B., Govender T., Maguire G.E., Soliman M.E., Kruger H.G. (2014). Integrated approach to structure-based enzymatic drug design: Molecular modeling, spectroscopy, and experimental bioactivity. Chem. Rev..

[15] Nam K., Shao Y., Major D.T., Wolf-Watz M. (2024). Perspectives on computational enzyme modeling: From mechanisms to design and drug development. ACS Omega.

[16] Cavatão F.G. (2024). A Base Molecular da Ativação da MC1R: Alterações Induzidas por Mutação na Dinâmica Estrutural. Master’s Thesis.

[17] Verkhivker G.M., Agajanian S., Kassab R., Krishnan K. (2022). Landscape-based protein stability analysis and network modeling of multiple conformational states of the SARS-CoV-2 spike D614G mutant: Conformational plasticity and frustration-induced allostery as energetic drivers of highly transmissible spike variants. J. Chem. Inf. Model..

[18] Ma B., Nussinov R. (2010). Enzyme dynamics point to stepwise conformational selection in catalysis. Curr. Opin. Chem. Biol..

[19] Wittmund M., Cadet F., Davari M.D. (2022). Learning epistasis and residue coevolution patterns: Current trends and future perspectives for advancing enzyme engineering. ACS Catal..

[20] Schell K. (2023). Alternative Active Site Confinement by Enforcing Substrate Pre-Organization in Cyclases. Ph.D. Dissertation.

[21] Black C., Huang H.-W., Cowan J. (1994). Biological coordination chemistry of magnesium, sodium, and potassium ions. Protein and nucleotide binding sites. Coord. Chem. Rev..

[22] Richard J.P. (2019). Protein flexibility and stiffness enable efficient enzymatic catalysis. J. Am. Chem. Soc..

[23] Klinman J.P. (2015). Dynamically achieved active site precision in enzyme catalysis. Acc. Chem. Res..

[24] da Fonseca A.M., Caluaco B.J., Madureira J.M.C., Cabongo S.Q., Gaieta E.M., Djata F., Colares R.P., Neto M.M., Fernandes C.F.C., Marinho G.S. (2024). Screening of potential inhibitors targeting the main protease structure of SARS-CoV-2 via molecular docking, and approach with molecular dynamics, RMSD, RMSF, H-bond, SASA and MMGBSA. Mol. Biotechnol..

[25] Gerlt J.A. (1987). Relationships between enzymatic catalysis and active site structure revealed by applications of site-directed mutagenesis. Chem. Rev..

[26] Skolnick J., Gao M., Zhou H., Singh S. (2021). AlphaFold 2: Why It Works and Its Implications for Understanding the Relationships of Protein Sequence, Structure, and Function. J. Chem. Inf. Model..

[27] Hou X., Du J., Zhang J., Du L., Fang H., Li M. (2013). How to improve docking accuracy of AutoDock4.2: A case study using different electrostatic potentials. J. Chem. Inf. Model..

[28] Lill M.A., Danielson M.L. (2010). Computer-aided drug design platform using PyMOL. J. Comput. Aided Mol. Des..

[29] Edmunds N.S., Genc A.G., Mcguffin L.J. (2024). Benchmarking of AlphaFold2 accuracy self-estimates as indicators of empirical model quality and ranking: A comparison with independent model quality assessment programmes. Bioinformatics.

[30] Allen J.R., Flanagan M., Pathak S., Emenecker S.K., Emenecker R.J., Strader L.C. (2025). An improved toolkit of gateway- and gibson assembly-compatible vectors for protoplast transfection and agrobacterium-mediated plant transformation. BMC Res. Notes.

[31] Awai S.K. (2004). Establishment of “Highly Efficient” Transformation Method of Saccharomyces Cerevisiae Based on Molecular Basis for Yeast’s Competence.

